# The role of a sequencing-based clinical intestinal screening test in patients at high-risk for *Clostridium difficile* and other pathogens: a case report

**DOI:** 10.1186/s13256-018-1919-1

**Published:** 2019-01-15

**Authors:** Maureen Hitschfeld, Elena Tovar, Sarah Gupta, Elisabeth M. Bik, Christina Palmer, Michael C. Hoaglin, Daniel E. Almonacid, Jessica Richman, Zachary S. Apte

**Affiliations:** 1 uBiome, San Francisco, CA USA; 2 University of California San Francisco, San Francisco, CA USA

**Keywords:** *Clostridium difficile*, Irritable bowel syndrome, Screening test, Gastrointestinal surgery, Comorbidity, Multimorbidity, IBS, CDI

## Abstract

**Background:**

Hospitalization and antibiotic treatment can put patients at high risk for *Clostridium difficile* infection, where a disturbance of the gut microbiome allows for *Clostridium difficile* proliferation and associated symptoms, including mild, moderate, or severe diarrhea. *Clostridium difficile *infection is challenging to treat, often recurrent, and leads to almost 30,000 annual deaths in the USA alone. Here we present a case where SmartGut™, an at-home, self-administered sequencing-based clinical intestinal screening test, was used to identify the presence of *Clostridium difficile* in a patient with worsening diarrhea. Identification of this pathogen and subsequent treatment led to a significant improvement in symptoms.

**Case presentation:**

The patient is a 29-year-old white woman with a history of severe irritable bowel syndrome with diarrhea, hemorrhoidectomy, and anal sphincterotomy complicated by a perianal fistula and perirectal abscesses that required extended courses of broad-spectrum antibiotics. In June 2016, she developed intermittent *Clostridium difficile* infections, which required continued antibiotic use. Months later she used an at-home, self-administered, intestinal microbial test, the first of which was negative for the presence of *Clostridium difficile*, but it detected the relative abundance of microbes associated with irritable bowel syndrome outside the healthy reference ranges. In the subsequent 2 months after the negative *Clostridium difficile* result, her gastrointestinal symptoms worsened dramatically. A second microbiome test resulted in a positive *Clostridium difficile* finding and continued abnormal microbial parameters, which led the treating physician to refer her to a gastroenterologist. Additional testing confirmed the presence of *Clostridium difficile* with a toxin-positive strain. She received treatment immediately and her gastrointestinal symptoms improved significantly over the next week.

**Conclusions:**

This case report suggests that more frequent DNA testing for *Clostridium difficile* infections may be indicated in patients that are at high-risk for *Clostridium difficile* infection, especially for patients with irritable bowel syndrome, and those who undergo gastrointestinal surgery and/or an extended antibiotic treatment. This report also shows that such testing could be effectively performed using at-home, self-administered sequencing-based clinical intestinal microbial screening tests. Further research is needed to investigate whether the observations reported here extrapolate to a larger cohort of patients.

## Background

The human gut microbiome consists of trillions of microorganisms that populate the human gut, and its genome is approximately 100 times larger than the human genome [1]. It is considered by some to act as an organ due to the number of physiological functions that it performs for the host [2]. Under normal conditions, commensal microbes and their hosts enjoy a symbiotic relationship. In addition, a greater diversity of microorganisms is generally an indicator for gut health, presumably because it offers greater resistance to perturbation [3, 4]. Intestinal dysbiosis refers to identifiable abnormality of the gut microbiota, either due to low microbial diversity, suboptimal levels of commensal beneficial microbes, excess of potentially harmful microorganisms, or presence of pathogenic microbes [5, 6]. Maladaptive alterations of the microbiome composition result in deficient functions, which are associated with various prevalent conditions, such as irritable bowel syndrome (IBS), inflammatory bowel disease (IBD), type 2 diabetes, prediabetes, obesity, non-alcoholic fatty liver disease, atherosclerosis, cardiovascular disease, kidney stones, among others [7–13]. Interestingly, the host interactions with the gut microbiome and its composition are not static, but rather dynamic and modifiable over time [14]. Diet, medication, and lifestyle are important determinants of microbial composition [15–17]. However, the largest and most immediate effects on microbial composition are related to the use of antibiotics, which may negatively affect the composition and function of the gut microbiota and consequently have an impact on patient well-being [18].

In the USA, IBS is the most common functional gastrointestinal (GI) disorder, with a reported prevalence between 10 and 25%; women exhibit prevalence rates 1.5-fold to 3-fold higher than men [19]. This chronic condition is characterized by abdominal pain associated with bowel dysfunction. Although IBS etiology is not completely understood, it is regarded as multifactorial. Contributing factors include intestinal motility, inflammation, genetics, immunology, psychology, and diet [20–22]. There are several subtypes of IBS, including IBS with constipation (IBS-C), IBS with diarrhea (IBS-D), mixed IBS (IBS-M), and unclassified (IBS-U) [23]. Initial treatment may include dietary and lifestyle changes. In particular, the low fermentable oligosaccharides, disaccharides, monosaccharides, and polyols (FODMAP) diet shows benefits in the management of IBS [24, 25]. Cognitive behavioral therapy has also been shown to be successful in managing IBS symptoms because anxiety and depression are commonly seen in patients with IBS [26]. Patients with more severe symptoms may also benefit from pharmacologic therapy to address predominant symptoms. A diagnosis of IBS is difficult to make. It has been estimated that up to 75% of cases of IBS go undiagnosed [27]. Studies show that patients with IBS have an underlying alteration of their gut microbiota composition [28–31]. For instance, it is known that abundance of the genus *Veillonella* is positively associated with IBS, while the relative abundances of genera *Alistipes, Bifidobacterium*, and *Lactobacillus* and the species *Collinsella aerofaciens* are inversely associated with IBS [16, 32–35].

*Clostridium difficile* is currently the leading cause of infectious nosocomial diarrhea in the USA; the incidence and severity of *C. difficile* infection (CDI) are increasing and are associated with increased health care costs [36]. *C. difficile* is an opportunistic pathogen, with toxigenic strains known to cause high levels of morbidity and mortality. There are a number of well-known risk factors that have been linked to CDI, including exposure to antibiotics and gastric acid suppressants, hospitalization, GI surgery, working in a health care setting, and the presence of underlying disease or immunosuppression [37, 38]. According to the Centers for Disease Control and Prevention, *C. difficile* was responsible for almost half a million infections and was associated with approximately 29,000 deaths in 2011 [39]. Toxicity from CDI is mediated by two exotoxins: toxin A and toxin B [40]. Colonization of the gut microbiota with *C. difficile* can be innocuous and asymptomatic; however, if the normal intestinal microbial architecture is disrupted, proliferation of opportunistic *C. difficile* may occur. There is evidence that CDI risk in *C. difficile* carriers is nine times higher than in non-carriers [41], and in healthy adult patient populations, as many as 15% of people may be colonized with *C. difficile*; prevalence rates appear to vary widely depending on the population [42].

There are limited data on what role CDI may play in patients with IBS. A cohort study showed that a new-onset of IBS is common after CDI. Infection duration, anxiety, and higher body mass index (BMI) were found to be associated with higher risk of the diagnosis of *C. difficile* in patients with post-infectious IBS [43]. Another study suggested that a subpopulation of patients with IBS in the absence of known risk factors for *C. difficile* may be susceptible and predisposed to CDI [44]. In this small study, *C. difficile* incidence among patients with IBS (5 out of 87) was higher than in the control group (1 out of 88), albeit not statistically significant, and the positive cases were found among all three IBS subtypes [44].

Here we present a case of a patient with a diagnosis of IBS-D, who used an at-home, self-administered, sequencing-based clinical intestinal microbial screening test (SmartGut™, uBiome Inc., San Francisco, USA) to identify the presence of *C. difficile* in the setting of multiple chronic conditions. Treatment for *C. difficile* led to resolution of her symptoms.

## Case presentation

This patient is a 29-year-old white woman from the USA with a medical history significant for severe IBS-D (diagnosed at age 12) and anxiety disorder. In July 2015, she presented with severe bleeding hemorrhoids secondary to IBS, which required hemorrhoidectomy and anal sphincterotomy in August 2015. The week before the surgery she developed pharyngitis and was treated with azithromycin, which resulted in mucousy diarrhea and abdominal discomfort. She tested negative for *C. difficile* antigen and toxins at that time.

A week after surgery, she developed a perirectal abscess that had formed at the site of the sphincterotomy and was prescribed orally administered ciprofloxacin. Despite moderate symptom improvement, in September 2015 she required an abscess incision and drainage procedure and Penrose drain insertion. Prior to the surgery she was given a single dose of clindamycin. An additional 2-week course of ciprofloxacin and metronidazole was then prescribed. In late September 2015 she was admitted to the hospital for two nights due to further complications related to the abscess and was then diagnosed as having a perianal fistula.

In November 2015, she was prescribed clindamycin for an episode of group C streptococcal-positive pharyngitis. In late November 2015, she was also diagnosed as having Ehlers–Danlos syndrome, which according to her medical record may partially explain the poor wound healing from the perirectal abscess. In December 2015, her fistula required an anus seton placement. She was treated with multiple courses of ciprofloxacin and metronidazole off and on from December 2015 to January 2016.

In January 2016, following up on her recurrent pharyngitis, she was diagnosed as having chronic tonsillitis which led to tonsillectomy. In February 2016, 2 weeks after the surgery she was prescribed clindamycin. At the beginning of March 2016, she was diagnosed as having bacterial vaginosis and was prescribed orally administered metronidazole. A week later she was diagnosed as having vaginal candidiasis and was prescribed orally administered fluconazole. In April 2016, she complained of dysuria and was prescribed ciprofloxacin. After 2 days, when urine analysis results came back negative, she was asked by her physician to stop the treatment.

In June 2016, she presented for follow-up with ongoing diarrhea and abdominal pain. She was diagnosed as having *C. difficile* diarrhea, her antigen and toxins laboratory results were indeterminate, and a toxigenic strain was confirmed by polymerase chain reaction (PCR). She was prescribed a 6-week course of orally administered vancomycin. After a week of treatment her symptoms worsened, and following discussion with her gastroenterologist her treatment was switched to a 2-week course of metronidazole. Hours later, she was admitted to the hospital for a 4-day period for colitis. Her *C. difficile* antigen and toxin test returned negative during her admission. She received intravenously administered metronidazole treatment during her hospitalization. Her symptoms improved during her hospital stay, with 1–2 soft bowel movements a day. At discharge her metronidazole course was stopped and she was again prescribed vancomycin, which she took for over a month. She continued to experience GI irregularity (3–5 bowel movements a day) beyond what she had experienced secondary to her IBS prior to her surgeries. In March 2017, she was prescribed rifaximin for 2 weeks to treat chronic diarrhea.

**Fig. 1:**
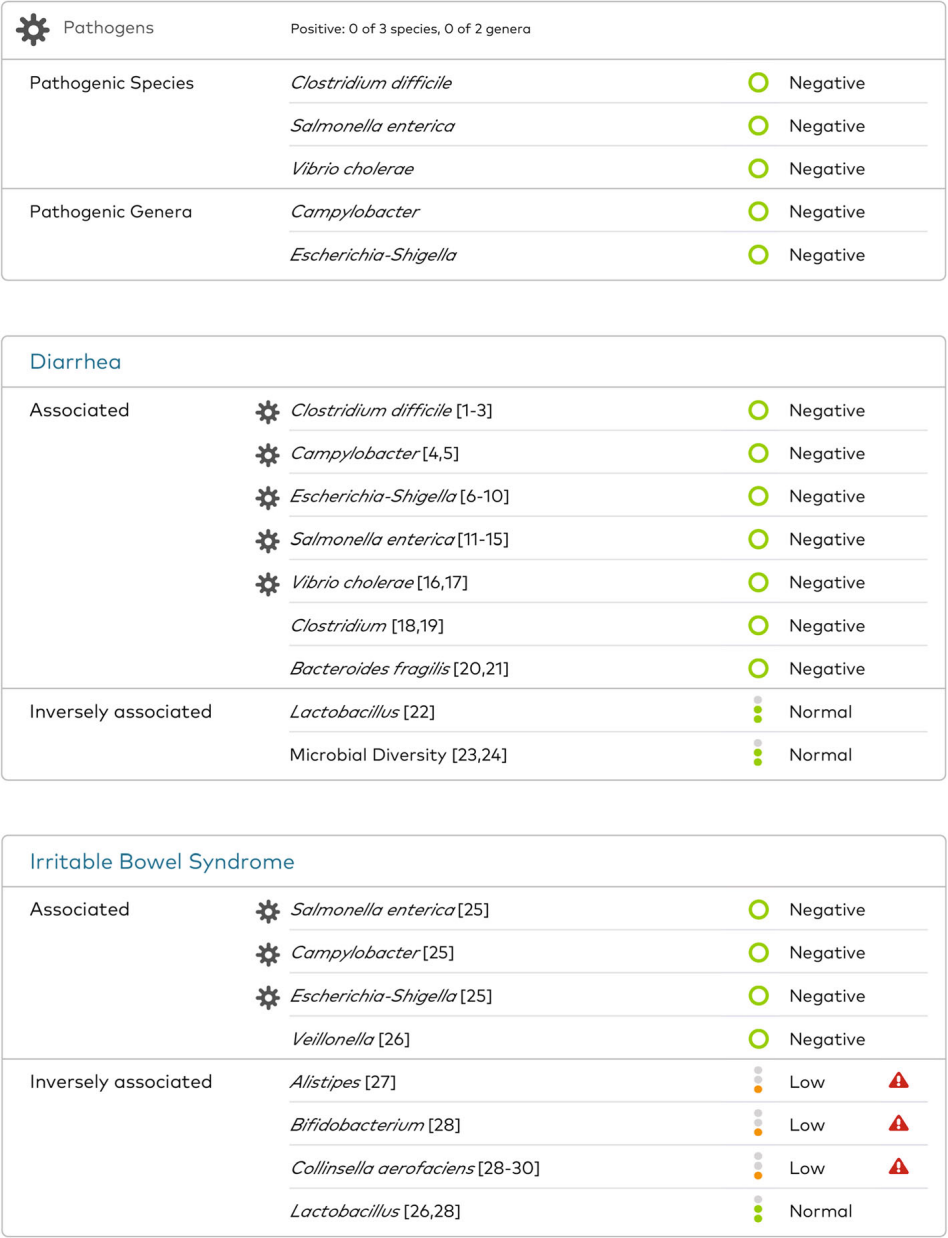
Selected results from the first SmartGut™ test report taken by the patient in November 2017 (*Clostridium difficile* negative). Three relevant parts of the clinical intestinal test SmartGut™ report generated after sample analysis are shown here: pathogens section, diarrhea from the infections section, and irritable bowel syndrome from the gut conditions section. The actual report is much more comprehensive and includes: other gut conditions such as inflammatory bowel disease, including ulcerative colitis and Crohn’s disease, and gastrointestinal symptoms; diet and lifestyle conditions, such as obesity, kidney stones, type 2 diabetes, non-alcoholic fatty liver disease, and prediabetes; and cardiovascular health conditions, such as atherosclerosis and cardiovascular disease, among others

In November 2017, she was prescribed a series of clinical intestinal tests (SmartGut™, uBiome Inc., San Francisco, USA) with the instructions to administer the test at home whenever she was experiencing a noticeable change of GI symptoms, then follow-up with her health care provider to discuss the results. This sequencing-based test requires that patients use a sterile swab to transfer a small amount of fecal material from toilet paper into a vial containing a lysis and stabilization buffer that preserves the microbial DNA for transport by mail back to the laboratory for processing, which involves DNA extraction, 16S ribosomal RNA (rRNA) gene amplification, and sequencing [45]. She first used this test in November 2017, about a month after completing a 2-week course of rifaximin. The results revealed a number of microbial organisms that were outside the healthy reference ranges, but she was negative for all pathogenic organisms included in the test, including *C. difficile* (Fig. 1)*.*

**Fig. 2:**
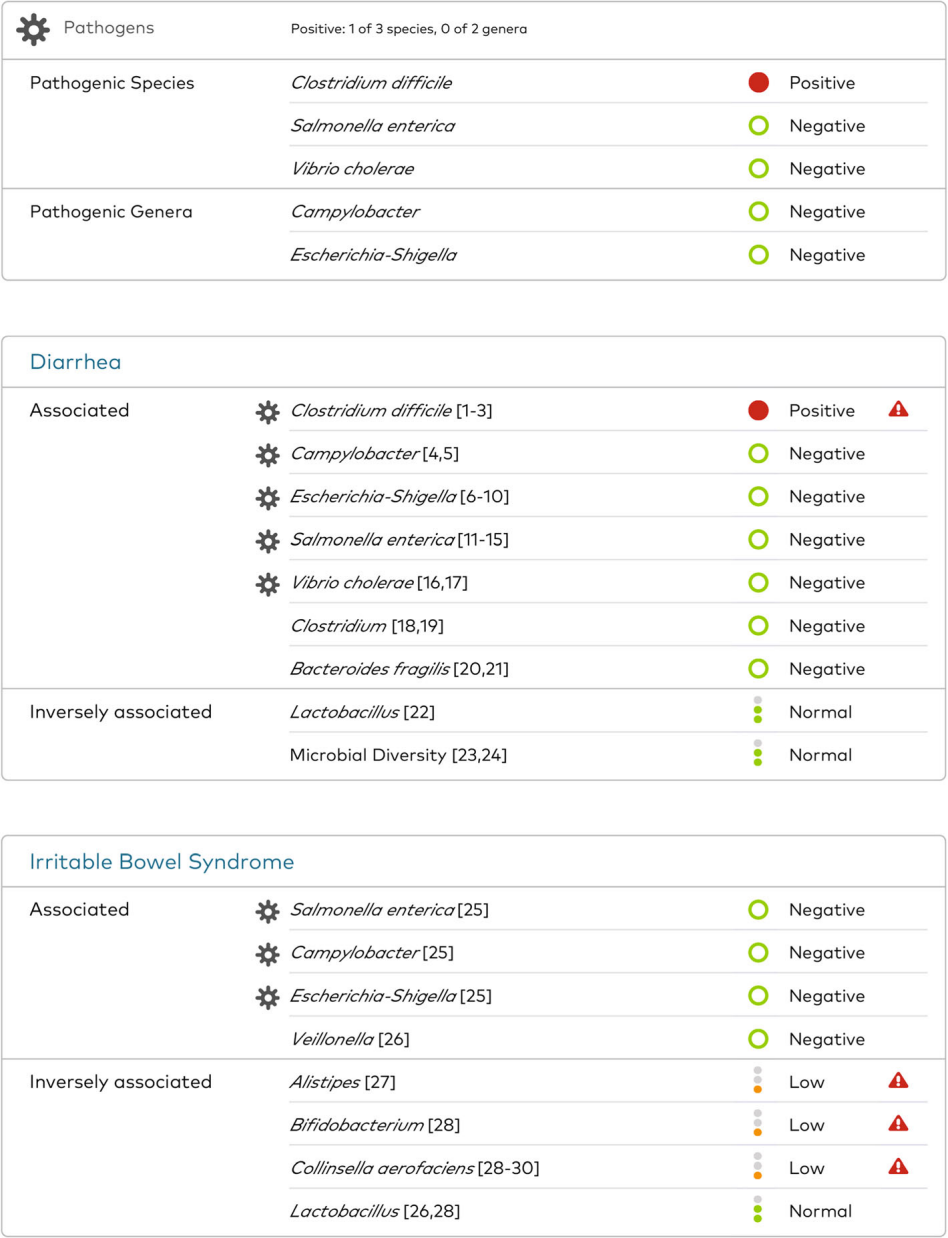
Selected results from the second SmartGut™ test report taken by the patient in January 2018 (*Clostridium difficile* positive)

Between November and December 2017, her GI symptoms worsened considerably; her daily bowel movements increased from 3–4 to 6–10, stool consistency became more mucous-like and gelatinous, and she was experiencing more pain with defecation. She re-tested with SmartGut™ test again in January 2018. Her results continued to reveal a number of microbial organisms outside the healthy range and, this time, her sample also indicated the presence of *C. difficile* (Fig. 2). She immediately contacted her primary care provider, who re-tested her for *C. difficile* and confirmed indeterminate CDI by antigen and toxins A and B. Additional PCR testing at a regional laboratory confirmed the sample was positive for a toxigenic *C. difficile* strain. As a result of testing, her clinician started her on fidaxomicin; her symptoms improved rapidly. By April 2018, she had returned to her baseline in regard to her IBS-related GI symptoms with no blood in her stools.

In addition, the second SmartGut™ sample was tested for toxins A and B by sequencing at uBiome Inc. laboratory in San Francisco, USA, which resulted positive for both and confirmed the toxigenic nature of the *C. difficile* strain.

## Discussion and conclusions

The patient had multiple risk factors for *C. difficile* colonization and overgrowth, such as several out-patient visits, hospitalizations, and previous GI surgery [46]. In this case, it is particularly important to note that repeat administration of combination and/or long-term broad-spectrum antibiotics may have contributed to changes in the patient’s intestinal microbiome, potentially contributing to *C. difficile* proliferation. During treatment with antibiotics, bacterial organisms that are susceptible to the antibiotics decrease in abundance or disappear, which potentially generates a microenvironment that enables *C. difficile* to thrive and increase in relative abundance, which could lead to the development of CDI [47–49]. Hence, it is recommended to prescribe antibiotics prudently. An example of a successful antibiotic stewardship intervention reducing CDIs in Scotland was avoiding the use of “4C” antibiotics: fluoroquinolones (for example, ciprofloxacin), clindamycin, co-amoxiclav, and third-generation cephalosporins, and using narrow-spectrum antibiotics instead (for example, fidaxomicin) [48]. Hence, reported new IBS treatment concepts involving the use of antibiotics need attention [50].

Importantly, this case study suggests that in high-risk populations, such as those repeatedly treated with antibiotics, there may be a need for more frequent and convenient testing for *C. difficile* and possibly for other GI pathogens as well. It makes sense that a self-administered, at-home, non-invasive test is an ideal adjunct solution for regular monitoring between scheduled appointments, particularly in patients who are already burdened by multiple visits to their health care providers or even by hospitalizations. Patients with known risk factors for CDI such as immunodeficiency, cystic fibrosis, and diabetes, or prolonged administration of antibiotics, proton pump inhibitors, and antidiarrheal medication [46] may need to be screened more often and may benefit from at-home testing.

Further investigation is greatly needed to understand the potential connection between the human intestinal microbiome, IBS, and the presence of *C. difficile*. The role of *C. difficile* in IBS and co-occurrence rates in particular would also benefit from further research. It remains to be determined if incomplete treatment of CDI might be associated with the development of IBS, or if ongoing IBS might increase the risk of acquiring *C. difficile*. Current clinical outcomes analyses using data from our uBiome citizen science cohort will further illuminate whether individuals diagnosed as having IBS have higher carriage rates of *C. difficile*, a finding previously reported in other studies [44]. Disturbance of the gut microbiome may therefore predispose patients with IBS to CDI and subsequent exacerbation of existing IBS symptoms due to the pathogen’s toxigenic nature [43].

While our product (that is, SmartGut™) is not intended in any way to replace standard of care or to function as a diagnostic tool for acute infection, the use of an at-home clinical microbial screening test for pathogenic and commensal bacteria associated with chronic conditions is a minimally invasive testing option that may provide clinicians and patients with unique information that could positively impact time to treatment, as well as improve patient outcomes.

## Abbreviations

BMI: Body mass index
CDI: *Clostridium difficile* infection
FODMAP: Fermentable oligosaccharides, disaccharides, monosaccharides, and polyols
GI: Gastrointestinal
IBD: Inflammatory bowel disease
IBS: Irritable bowel syndrome
IBS-C: Irritable bowel syndrome with constipation
IBS-D: Irritable bowel syndrome with diarrhea
IBS-M: Mixed irritable bowel syndrome
IBS-U: Unclassified irritable bowel syndrome
PCR: Polymerase chain reaction
rRNA: Ribosomal RNA
